# Dataset for rapid estimation of femoral neck loading during gait and dynamic exercises

**DOI:** 10.1038/s41597-026-07429-3

**Published:** 2026-05-22

**Authors:** David Hollinger, Zainab Altai, Jason Moran, Xiaojun Zhai, Andrew Phillips, Qichang Mei, Bernard X. W. Liew

**Affiliations:** 1 School of Computer Science and Electronics Engineering, University of Essex, Colchester, Essex UK; 2 School of Sport, Rehabilitation, and Exercise Sciences, University of Essex, Colchester, Essex UK; 3 Department of AI in Preventive Medicine, King’s College London, London, UK; 4 Department of Civil and Environmental Engineering, Imperial College London, London, UK; 5 Faculty of Sports Science, Ningbo University, Ningbo, China; 6 Auckland Bioengineering Institute, The University of Auckland, Auckland, New Zealand

**Keywords:** Bone quality and biomechanics, Medical research

## Abstract

Medical intervention related to musculoskeletal (MSK) and orthopaedic conditions often requires an understanding of the loads experienced at the joints. A wide spectrum of exercise types and intensity levels can alter joint loading which is useful for clinical interventions related to mobility, balance, risk of fragility fracture, and post-surgical recovery (e.g., total hip arthroplasty). Currently, there is a lack of publicly available datasets on bone loading across varied intensities during gait and dynamic exercises (e.g., running, jumping, and hopping). In order to broaden the understanding of load-based physical therapy, we present a dataset comprising 40 healthy participants performing walking, running, countermovement jumps, squat jumps, unilateral hopping, and bilateral hopping across three intensity levels (high, moderate, and low). This dataset includes inertial measurement signals (IMU), joint kinematics and kinetics from MSK modelling in OpenSim, and tensile and compressive femoral neck strains from finite element analysis. Overall, the multimodal signals in this dataset support medical research and clinical interventions to enhance bone health, reduce fracture risk, and accelerate the rehabilitation process.

## Background & Summary

Exercise triggers a mechanical loading response across musculoskeletal (MSK) joints which influences bone health and subsequently the risk of bone fractures^1^. For example, increased loading at the hip joint (e.g., femoral neck) can improve the osteogenic response and so enhance bone mineral density which, in turn, reduces the risk of hip fractures^2,3^. However, the loading profile can vary based on the type and intensity of the exercise^3^. Since load-based exercises can induce a wide range of peak strains, from low (e.g., slow walking) to high (e.g., unilateral hopping)^2^, clinicians can prescribe exercises to meet a patient’s loading needs for optimal bone health. Therefore, clinical strategies aimed at reducing fracture risk and enhancing MSK health often modulate load-based exercises during rehabilitation^4^.

Although load-based monitoring can enhance joint health during rehabilitation, certain methods to monitor joint strain, such as sensor implantation, are invasive, costly and impractical^5^. Currently, wearable sensors and motion capture technology can measure limb motion and ground reaction forces but MSK modelling combined with finite element (FE) analysis is needed for non-invasive strain estimation^6–8^. For example, computational modelling of the femoral neck characterized by mechanical strains can compare osteogenic potential across a variety of different exercises^9^. This approach facilitates the possibility of quantifying and comparing bone-loading profiles across various tasks. As a result, clinicians can adjust therapy exercises to optimize patient-specific bone-loading outcomes^8^.

Although various gait datasets exist^10,11^, there are no comprehensive open source datasets combining gait and dynamic exercises with bone strain, motion capture, and IMU signals. As illustrated in Table 1, published open source datasets typically comprising walking^12–15^ and running^16^ across varying speeds and inclines exist, but lack dynamic exercises for a more comprehensive approach to bone health. Dynamic exercises are especially important for patients who may need to incorporate movements with a greater load response at the hip. Keast *et al*. and Sinclair & Taylor shared datasets which included tibial strain from FE modelling but they were limited to running activity only^17,18^. Rico-Garcia *et al*. shared a jumping dataset but the data sources were limited to motion capture and IMU signals^19^. Scherpereel *et al*. provided a biomechanical dataset during gait and dynamic tasks (e.g., jumping, lunges, and squatting) during cyclic and non-cyclic activities^11^. However, their dataset lacks strain data which limits therapeutic applications aimed at enhancing bone mineral density.

**Table 1:** Comparison of publicly available datasets with gait and dynamic movements.

Metadata			Gait		Dynamic Movements				Data Source		
Study (Year)	Participants	Age (yrs)	Walking	Running	Bilateral hopping	Unilateral hopping	Counter jumping	Squat jumping	Mocap	IMU	Strain
Moore *et al*.^12^	15 (4 F, 11 M)	24 ± 4	0.8 m/s, 1.2 m/s, 1.6 m/s	—	—	—	—	—	√	—	—
Fukuchi *et al*.^16^	28 (healthy runners)	19–51 range	—	2.5 m/s, 3.5 m/s, 4.5 m/s (treadmill)	—	—	—	—	√	—	—
Hu *et al*.^15^	10 (7 M, 3|F)	25.5 ± 2.0	Level & stair walking; 10° incline/decline	—	—	—	—	—	√	√	—
Lencioni *et al*.^13^	50 (25 M, 25 F)	6–72 range	Self-selected speed; accel/deceleration trials	—	—	—	—	—	√	—	—
Camargo *et al*.^14^	22 (13 M, 9 F)	21 ± 3.4	0.5–1.85 m/s (level-ground, incremented)	—	—	—	—	—	√	√	—
Scherpereel *et al*.^11^	12 (7 M, 5 F)	21.8 ± 3.2	0.4 m/s, 0.6 m/s, 1.2 m/s, 1.8 m/s	2.0 m/s, 2.5 m/s	—	—	√ (vertical jumps)	—	√	√	—
Keast *et al*.^17^	30 cadavers (20 M, 10 F)	28.7 ± 6.7	√	√	—	—	—	—	—	—	Tibial strain
Sinclair & Taylor^18^	15 M	18–40 range	—	√ (4.0 m/s treadmill)	—	—	—	—	√	—	Tibial strain
Rico-Garcia *et al*.^19^	4 healthy adults	20–30 range	—	—	—	—	√ (vertical jumps)	—	√	√	—
**Proposed Dataset**	**40** (**20 M,** **20 F**)	**19**–**69 range**	**3 speeds** (**slow,** **moderate,** **fast**)	**3 speeds** (**slow,** **moderate,** **fast**)	**15 s bouts @ ~2**.**2 Hz,** **2**.**6 Hz,** **and 3**.**0 Hz**	**15 s bouts @ ~2**.**2 Hz,** **2**.**6 Hz,** **and 3**.**0 Hz**	**50%,** **75%,** **100% max effort**	**50%,** **75%,** **100% max effort**	√	√	**Femoral neck strain**

The proposed dataset is emboldened at the bottom. Abbreviations: mocap = motion capture; IMU = inertial measurement unit; √ denotes the inclusion of the corresponding task or data source; “—” denotes not included.

Despite the need for diverse exercises in rehabilitation training to support flexible osteogenic adaptation, there remains a significant gap in publicly available datasets that quantify bone loading across a broad range of movements and intensities. To address this gap, we present a comprehensive dataset comprised of motion capture, IMU signals and strain data during varied-intensity gait and dynamic exercises from 40 healthy adults aged 18–70 years. Participants performed six gait and dynamic tasks (e.g., walking, running, countermovement jumps, squat jumps, unilateral hopping, and bilateral hopping) across three intensity levels (low, medium, and high). The dataset includes time-normalised 3D IMU signals (acceleration and gyroscope), ground reaction forces, joint angles, joint moments, joint reaction forces of the hip, knee and ankle estimated by MSK model, and model-derived estimates of femoral neck principal tensile and compressive strains (*ε*_1_ and *ε*_3_) calculated from FE analysis. The tensile and compressive strains at the hip in this dataset reflect various magnitudes of loading conditions acting on the femoral neck. This is important for understanding how mechanical stimuli affect bone adaptation, fracture risk and post-surgical environments (e.g., total hip replacement).

The proposed dataset presents a novel combination of data sources from motion capture (e.g., c3d and cmz files), MSK modelling, FE modelling and inertial wearable sensors. This multimodal and comprehensive dataset allows scientists and clinicians to rapidly estimate joint load across exercises. As a result, practitioners can quickly quantify *in vivo* tissue loads outside the traditional laboratory and compare them to normative values. Overall, this extensive dataset allows for quick access to joint loading profiles during exercise and enables personalized exercise programs to enhance bone health.

## Methods

### Participants

Forty (20 males and 20 females) healthy participants were recruited with an age range of 18 to 70 years old (mean ± 1 SD: age of 40.3 ± 13.1 years; height 1.71 ± 0.08 m; and body mass 68.44 ± 11.67 kg). All participants were free from any lower-limb joint surgeries, free from any lower-limb injuries and pain within the past 12 months, and from the point of recruitment. All participants provided written informed consent approved by the University of Essex Faculty of Science & Health Ethics Subcommittee (ETH2021-160 1155).

### Instrumentation

**Fig. 1:**
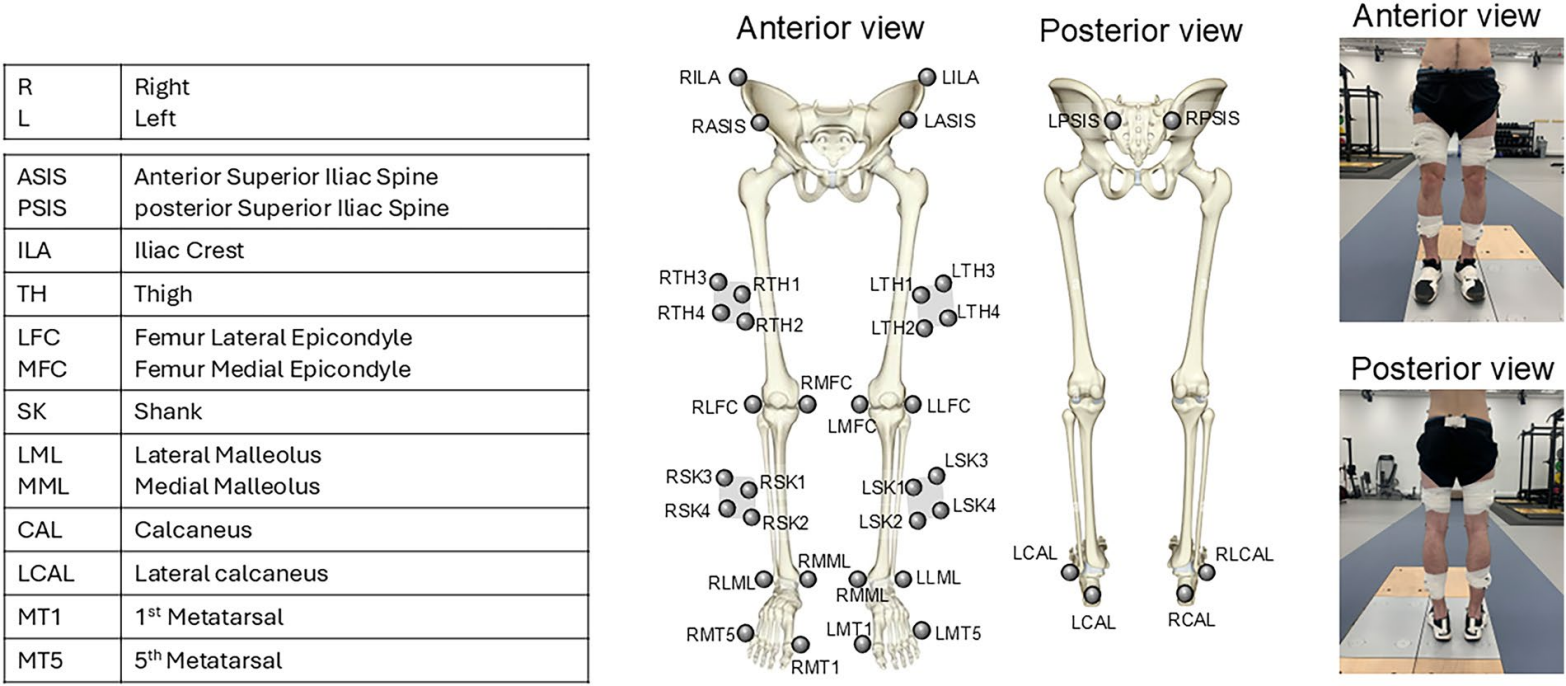
Marker placement and setup used for data collection.

Data collection used a 14-camera 3D motion capture setup (Vicon Ltd., Oxford, UK, 200 Hz). As shown in Fig. 1, thirty-eight retro-reflective markers were attached to the lower body. Twenty-two individual markers were placed on anatomical landmarks (left and right superior iliac spines, anterior superior iliac spines and posterior superior iliac spines, medial and lateral femoral condyles, medial and lateral malleoli, lateral and posterior aspects of the calcaneus, the first and fifth metatarsals). Tracking clusters consisting of four markers were attached to the distal lateral aspect of the thigh and the shank. Ground reaction forces were collected using two adjacent ground-embedded force platforms (Kistler, Winterthur, Switzerland, 2000 Hz).

**Fig. 2:**
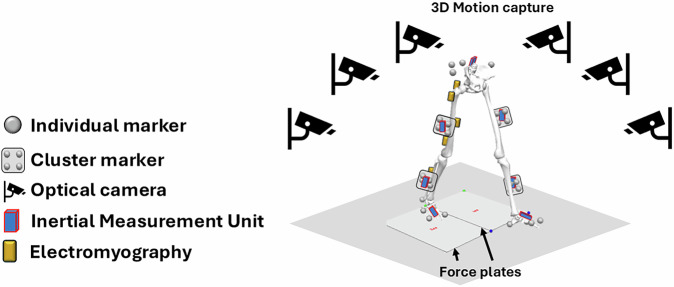
IMU placement and motion capture setup used for data collection.

As shown in Fig. 2, seven Blue Trident IMU sensors (Vicon Motion Systems Ltd, Oxford, UK, 225 Hz) were placed on the lower body segments of the left and right sides: posterior trunk, lateral shank, lateral thigh, and foot. For each IMU sensor, accelerations and gyroscope data were recorded in the three planes of motion; sagittal, coronal (frontal), and transverse planes (represented by three axes x, y, and z). A Vicon Lock system (Lock Lab/Lock Studio) was used to synchronise all data collection devices, including motion capture cameras, IMU sensors, and force plates. This system provides hardware-based triggering and a shared time base across devices to ensure sub-frame temporal alignment of all recorded signals during data acquisition. As synchronization was performed within the Vicon system using a common hardware trigger, no measurable delay was observed across devices.

### Experimental Design

As shown in Table 2, participants performed three successful trials of walking, running, countermovement jump, squat jump, unilateral hopping, bilateral hopping at three self-reported intensity levels (maximum, medium or intermediate, and minimum). Intensity levels were defined using self-selected effort levels to capture natural movement strategies across individuals and to reflect how these activities are typically performed in real-life settings. For hopping tasks, movement cadence was controlled using a metronome to standardize hopping frequency across participants. Walking and running trials were performed on a 20-meter flat runway and were included only when the participant’s dominant foot made full contact with the force plate without observable evidence of modified or stuttering gait. Countermovement jumping and squat jumping were performed at three intensity levels (e.g., minimum, medium, and maximum effort corresponding to 50%, 75%, and 100% maximum effort). For bilateral hopping and unilateral hopping trials, participants were asked to hop for 10 seconds and match their hopping speed with a metronome beat frequency of 2.2 Hz, 2.6 Hz, or 3.0 Hz, which represented minimum, medium, and maximum effort levels. Unilateral hopping was performed on the participant’s dominant limb. Limb dominance for each participant is reported in SubCharacteristics.xlsx in the dataset on Figshare^20^. Table 2 summarizes the start and end of each task, the number of repetitions, and the prescribed intensity levels. Further methodological details for each are described in a separate publication^2^.

**Table 2:** List of tasks, starting and ending criteria, number of repetitions, and intensity levels.

Task	Start	End	Reps	Intensity level
Walking	Heel strike of the dominant limb	Toe off of the dominant limb	3	Self-determined
Running	Heel strike of the dominant limb	Toe off of the dominant limb	3	Self-determined (Natural)
Running	Heel strike of the dominant limb	Toe off of the dominant limb	3	Moderate
Running	Heel strike of the dominant limb	Toe off of the dominant limb	3	Fast
Countermovement Jumping	Initial stand before take-off	Initial take-off	3	50% max effort at self-determined depth
Countermovement Jumping	Initial stand before take-off	Initial take-off	3	75% max effort at self-determined depth
Countermovement Jumping	Initial stand before take-off	Initial take-off	3	100% max effort at self-determined depth
Squat Jumping	Lowest pelvis position before take-off	Initial take-off	3	50% max effort at self-determined depth
Squat Jumping	Lowest pelvis position before take-off	Initial take-off	3	75% max effort at self-determined depth
Squat Jumping	Lowest pelvis position before take-off	Initial take-off	3	100% max effort at self-determined depth
Unilateral Hopping	Foot on force plate	Foot off force plate	3	2.2 hops/sec
Unilateral Hopping	Foot on force plate	Foot off force plate	3	2.6 hops/sec
Unilateral Hopping	Foot on force plate	Foot off force plate	3	3.0 hops/sec
Bilateral Hopping	Foot on force plate	Foot off force plate	3	2.2 hops/sec
Bilateral Hopping	Foot on force plate	Foot off force plate	3	2.6 hops/sec
Bilateral Hopping	Foot on force plate	Foot off force plate	3	3.0 hops/sec

### Data processing and normalisation

Marker trajectories were lowpass filtered at 18 Hz with a zero-lag second order low-pass Butterworth while force plate data were lowpass filtered at 50 Hz. IMU signals were filtered at 200 Hz using a 2nd order low-pass Butterworth^21,22^. Following data acquisition, custom MATLAB scripts were used for data processing. Trials were time normalized to 101 data points, representing 0–100% of the movement cycle, following standard practice of biomechanical data to ensure consistent comparison across trials and participants^23^. Additionally, the data were normalised to participant mass (kg). All joint moments were expressed in a participant-specific anatomical coordinate system following a Cardan (Euler) rotation sequence and the right-hand rule. The rotation order was Y–X–Z, with the anteroposterior (y) axis representing the sagittal plane flexion/extension moment, the mediolateral (x) axis representing the frontal plane abduction/adduction moment, and the longitudinal (z) axis representing the transverse plane internal/external rotation moment. To ensure inter-limb consistency, joint moments for left-limb dominant participants were mirrored about the sagittal plane, such that the y- and z-axis components corresponded anatomically to those of the right limb.

### Trial segmentation

**Fig. 3:**
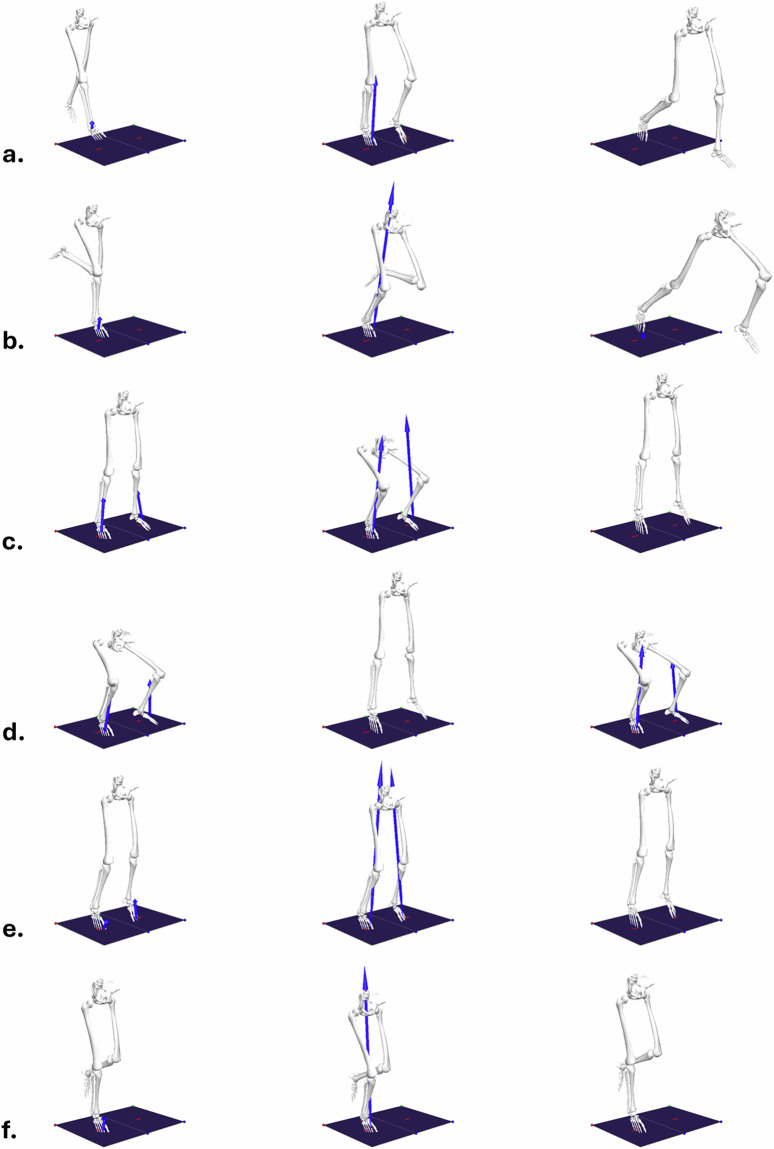
Visual representation of the segmented phases (start, middle, and end) for each task: (**a**) walking, (**b**) running, (**c**) countermovement jumping, (**d**) squat jumping, (**e**) bilateral hopping, and (**f**) unilateral hopping. Segmentation was based on predefined biomechanical events (e.g., heel strike, toe-off, lowest position of pelvis, and take-off), which were used to define the time window for analysis.

Trials were then segmented based on events detected on the participant’s dominant side. Gait events were defined by a vertical ground reaction force (vGRF) threshold of 30 N. Event labels were created for systematic segmentation. Figure 3 shows the visual representation of the trial segmentation performed using Visual 3D (C-motion Inc., Germantown, MD, USA) as follows: walking and running from heel strike to toe-off of the same foot, countermovement jump from the initial stand just before the take-off to initial take-off, squat jump from the lowest position of the pelvis just before the take-off to the lowest position of the pelvis after landing, and for bilateral and unilateral hopping from the foot on to foot off the force plate of the same leg. The time of interest for all trials was defined using the dominant side of the participant. In order to match the number of trials across dynamic tasks, only the fourth hop of each hopping trial was included in the dataset.

### Joint angles from Visual3D

**Fig. 4:**
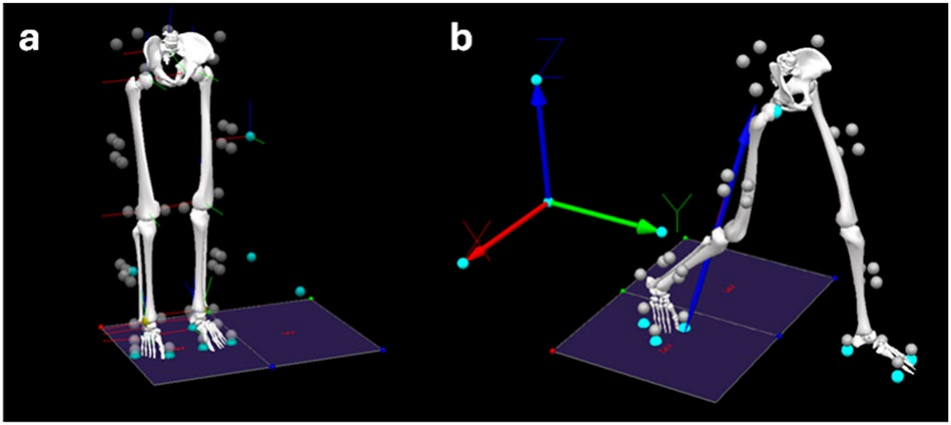
Visual3D model with a participant-centred coordinate system. (**a**) The static pose of the model shows the placement of markers on each segment and their corresponding anatomical coordinate axes. The x-, y-, and z-axes correspond to the medial-lateral, anterior-posterior, and inferior-superior directions, respectively. A participant-centred coordinate system of positive x, y, and z axes pointing laterally to the right side, anterior, and superior was used following the right-hand rule. (**b**) This anatomical coordinate system was used during dynamic trials. All motion capture and IMU data were processed within this anatomical reference frame.

Visual3D (C-Motion, USA) was used for preprocessing of the motion capture data, including marker trajectory filtering, event detection, and calculation of joint kinematics from the recorded c3d files. The processed marker trajectories and ground reaction forces (GRF) were subsequently exported to OpenSim for MSK modelling, where muscle forces and joint reaction forces were estimated. This workflow allowed robust experimental data processing in Visual3D while utilising OpenSim’s capabilities for MSK simulation. Furthermore, our c3d and OpenSim files allow users to perform MSK modeling in either OpenSim or Visual3D so users can compare the effects of different joint constraints and optimization strategies. The Visual3D six degrees of freedom (DOF) algorithm was used for segmental optimization to compute 3D joint angles and moments at the bilateral ankle, knee, and hip. The moments (y and z-direction) of the left lower-limb were flipped with left limb dominant subjects (e.g., subj05, subj23, subj24, and subj40). Joint angles were calculated using the Cardan rotation sequence of Y–X–Z described above. The Visual3D model consisted of seven segments (pelvis, bilateral thighs, shanks, and feet). Joint kinematics were computed using a 6 DOF (3 rotational and 3 translational) model at the ankle, knee, and hip joints (Fig. 4).

### Musculoskeletal modelling and inverse dynamics

**Fig. 5:**
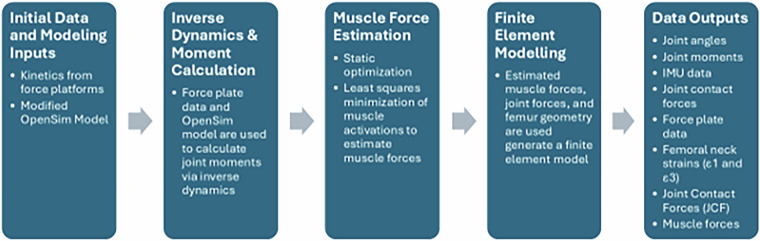
Workflow from initial data and model inputs to corresponding output data.

OpenSim was used to perform inverse dynamics and static optimization, while femoral neck strain data were derived using a standardized pipeline that combined MSK models and subject-specific morphing of a generic femoral model to simulate internal bone loading using ANSYS software. Kinetics from the force platforms and the resulting joint moments were calculated using a modified generic OpenSim gait2392 model^24^. Static optimisation was performed using sum of squares minimisation of muscle activations to estimate muscle forces. Details on the modified MSK model can be found in Altai *et al*.^2^. Briefly, this model removed the torso and corresponding muscles and incorporated 13 body segments, 18 DOF, and 86 Hill-type musculotendon actuators (Table 3). As shown in Fig. 5, force plate data, inverse dynamics (ID), joint contact forces (JCF) and muscle forces are expressed in Newtons (N). All MSK modelling outputs are reported from the dominant side, while Visual 3D, wearable sensors, and force plate data are reported for both sides. This resulted in IMU, ID and force plate (FP) data on both dominant and non-dominant sides while *ε*_1_, *ε*_3_, JCF, and muscle forces are reported only on the dominant side. Since left and right moments were reported, kinematic data from both sides were reported as well. The biomechanical data followed the standard 3D coordinate convention following the right-hand rule, where X = medial lateral axis (positive pointing lateral) measured flexion-extension, Y = anteroposterior axis (positive pointing anterior) measured abduction-adduction, and Z = Vertical axis (positive pointing up) measured axial rotation. Joint moments were expressed in a joint coordinate system defined relative to the proximal anatomical segment.

**Table 3:** Overview of data fields for each MATLAB file per participant.

MATLAB Struct Field	Original Collection Frequency (Hz)	Post-Processing	Units	Data Source	Description	Number of variables	Example Variable Names
N/A (angles.csv)	200	Time normalised to 101 data points	Degrees	Angles	3D joint angles for bilateral ankle, knee, and hip	18	L_Ankle_Angle_X, LAnkle_Angle_Y, L_Ankle_Angle_Z,… R_Hip_Angle_Z
IMU	225	Time-normalised to 101 data points	Acceleration (degrees/second^2^) Angular Velocity (degrees/second)	IMU	3D accelerometer and gyroscope at the pelvis, thighs, shanks, and feet	42	Accel.x_PEL, acc.y_RTH, … gyro.z_LFT
ID	N/A	Time normalised to 101 data points	Newton-meters/ kilogram	Inverse Dynamics	3D joint moments for bilateral ankle, knee, and hip	18	R_Hip_Moment_x, L_Knee_Moment_y, … R_Ankle_Moment_z
Force Plate	2000	Time normalised to 101 data points	Newtons	Ground Reaction Forces	GRF vectors for two force plates (XYZ components)	6	FP1_x, FP1_y, FP1_z; FP2_x, FP2_y, FP2_z
e1e3	N/A	Sampled at every 10^th^ point from a time normalised set of 101 data points	Unitless	Strain	Bone strain from FE analysis; provided at every 10^th^ point of time-normalised trial	2	e1, e3
JCF	N/A	Time normalised to 101 data points	Newtons	Joint contact forces	3D contact forces at the ankle, knee, and hip (dominant side)	8	JCFhip_fz, JCFknee_fy, JCFankle_fx
MuscleF	N/A	Time normalised to 101 data points	Newtons	Muscle forces	3D force vectors for lower limb muscles (dominant side)	77	glut_max1_r_Fx, psoas_r_Fy, vas_lat_r_Fz, bifemsh_r_Fz, iliacus_r_Fx, …

### Finite element modelling

Femoral neck strain data were computed from FE analysis (ANSYS, SpaceClaim 2023R1, PA, USA) for the first and third principal strains (*ε*_1_, *ε*_3_) which is a proxy for osteogenic potential^25^. Since personalised medical images were not available, the three-dimensional geometry of the full femur model for each participant was created using open-source Musculoskeletal Atlas Project (MAP) Client software^26^. A generic whole lower body shape model was registered to the marker set defined in the static trial to create the atlas femur mesh, which was then morphed into the femoral landmarks according to a femur statistical shape model^27^.

Although the finite element analysis focused on the femur, the initial registration was performed using a generic whole lower-body statistical shape model from the Victorian Institute of Forensic Medicine (Melbourne, VIC, Australia). The model was first registered to the participant-specific marker set obtained during the static calibration trial to ensure anatomical alignment of the pelvis and lower limb segments. Among all cases, the highest mean root-mean-squared (RMS) distance between correspondent points on the actual and registered models was 3.8 ± 2.7 mm. Subsequently, the femur geometry was reconstructed from anatomical landmarks derived from the motion capture data following the method described by Zhang *et al*.^26^. The atlas femur mesh was morphed to the participant-specific femoral landmarks using a femur statistical shape model^27^. For each femur, the RMS distance between data points and their closest point on the fitted mesh was calculated. Depending on the region of femur, the mean RMS distance was between 0.2 ± 0.09 mm and 0.71 ± 0.25 mm. This workflow preserves the pelvis–femur spatial relationship established during the whole-body registration while enabling subject-specific femur geometry for finite element analysis. The final morphed mesh quality was considered acceptable when the location of the muscle force application point, calculated from the MSK model, was within 3 mm of the external surface of the FE mesh (i.e., less than the maximum element size). This criterion ensured that the moment arms of the applied muscle forces were accurately represented and that no artificial increase or reduction in the resulting force moments was introduced. Homogeneous linear elastic isotropic material properties were defined for the bone with an elastic modulus of 18.6 MPa and a 0.3 Poisson’s ratio^28^.

The generated surface meshes for each participant were imported into ANSYS to generate 3D solid geometries. Femur models were then meshed using 10-nodes tetrahedral elements in ANSYS (ICEM CFD 2023R1, PA, USA) with an average element size of 3 mm^7,29^. While this simplification may risk overestimating stiffness in regions such as the femoral neck, where cortical bone is relatively thick, it provides a practical balance between computational efficiency and model robustness. Moreover, previous work^27^ has demonstrated that statistical modelling approaches can effectively account for cortical thickness variation, supporting the validity of adopting homogeneous properties in this context. This approach also facilitates comparability across studies by minimizing variability introduced through heterogeneous property assignment.

Muscle forces estimated from the MSK model were applied to the external surface of the femur as point loads in the FE model. Muscle attachment locations were used to determine the application points, which were mapped to the nearest surface mesh nodes. In all but three cases, the distance between the estimated application point and the nearest mesh node was less than the element size (3 mm); these three participants (Subj16, Subj29, and Subj 32) were excluded, resulting in a final cohort of 37. These participants were excluded as part of a quality control step to ensure accurate mapping of muscle force application points onto the finite element mesh. Distances exceeding the mesh element size ( >3 mm) may introduce errors in force application and resulting strain estimates. To prevent rigid body motion, the distal femur was kinematically constrained: the medial condyle’s most distal node was fixed in all directions; the lateral condyle’s node was constrained in the anterior–posterior and vertical directions; and a third node in the patellar groove was constrained anterior–posteriorly. These boundary conditions were selected to replicate the dominant joint movements in the studied exercises (e.g., hip/knee flexion-extension, rotation, and hip abduction–adduction). Due to the high computational cost, FE simulations were run at every 10^th^ timesteps per trial. At each timestep, peak first and third principal strains at the femoral neck were averaged over a 3 mm radius to minimize local effects, and their spatial locations were recorded^30^. Simulations were performed using ANSYS Mechanical (APDL 2023R1, PA, USA), with a typical runtime of one minute per timestep.

## Data Records

**Fig. 6:**
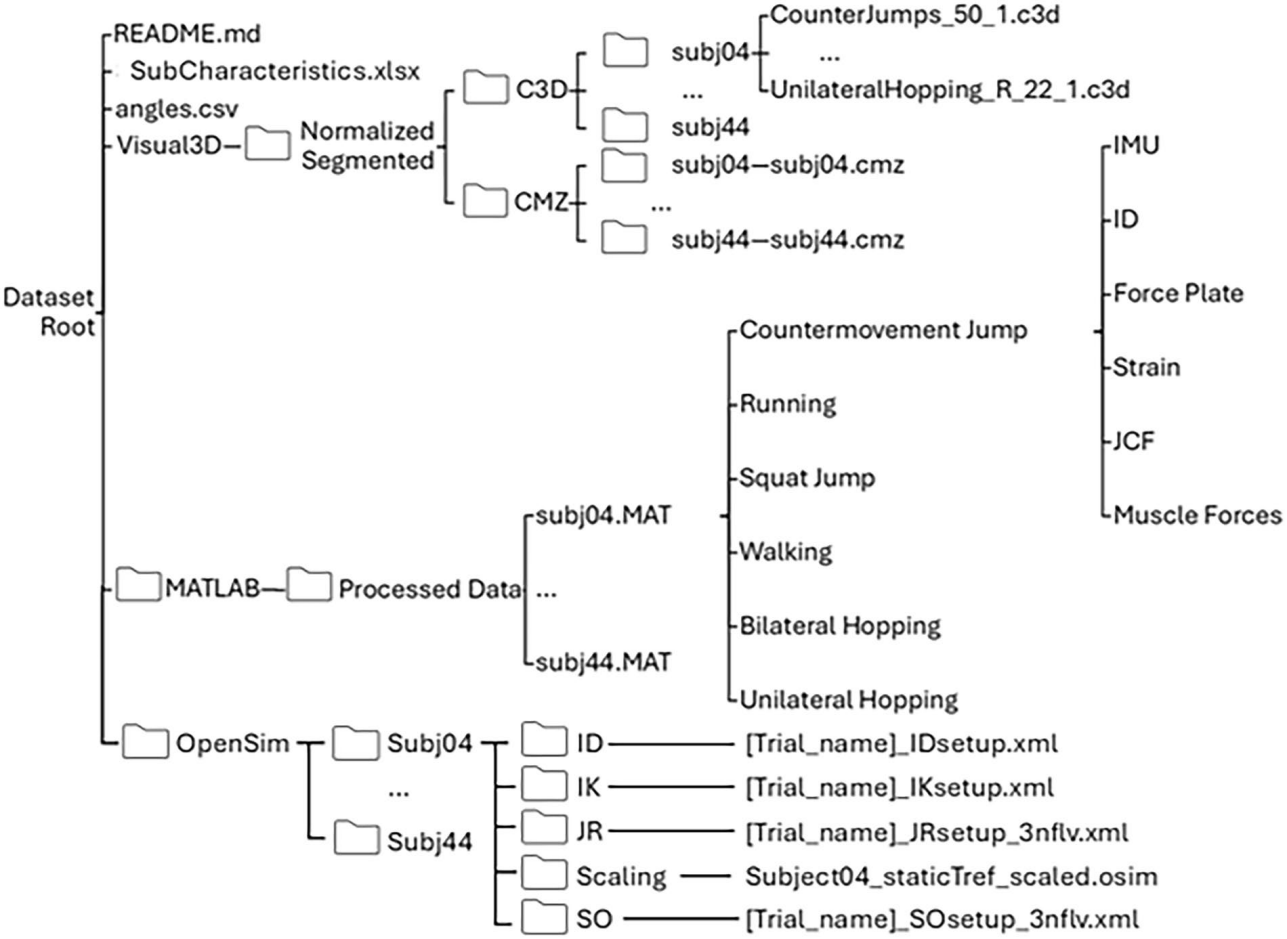
File structure of the relevant data sources.

The dataset can be downloaded from Figshare at: 10.6084/m9.figshare.30225739. The proposed dataset was stored in a “Processed Data” folder containing csv and MATLAB files (Fig. 6). The two Excel files (e.g., SubCharacteristics.xlsx and angles.csv) show demographic information and joint angles for all participants. Each participant has a corresponding MATLAB file comprised of subject-specific data (e.g., subj04.mat, subj05.mat, … subj44.mat). Originally, data were collected from 44 participants. Subjects 1–3 were excluded as they were part of pilot testing used to refine the experimental protocol and were therefore not included in the final analysis. Participant 7 was excluded due to substantial data quality issues, including missing marker trajectories and IMU sensor dropouts during the data collection session. Consequently, the final study cohort consisted of the remaining participants with complete and reliable datasets.

Each MATLAB file contains a MATLAB struct with the fields listed in Table 3. Filenames follow a structured convention based on the activity type, task parameter (e.g., limb, intensity, or frequency), and trial number. For example, UnilateralHopping_R_22_1.c3d represents unilateral hopping performed on the dominant right limb (R) at 2.2 Hz (22) for trial 1. Jump trials are labelled as SquatJumps_[IntensityLevel]_[TrialNumber] and CounterJumps_[IntensityLevel]_[TrialNumber], where intensity levels correspond to 50 (low), 75 (moderate), and 100 (maximal) effort. Walking trials follow Walking_[TrialNumber], while running trials follow Running_[IntensityLevel]_[TrialNumber] (Natural, Moderate, or Fast). For walking and running, trials missing the force plate were discarded; therefore, trial numbers may not always be sequential. Subject 9, 11, and 14 completed two hopping trials instead of three because they found it too difficult or tiring to continue for the required time period. As a result, the hopping trials for these participants are labelled trial 1, trial 2, and trial 1, respectively.

## Technical Validation

### Ground reaction forces, joint contact forces, and strains

**Fig. 7:**
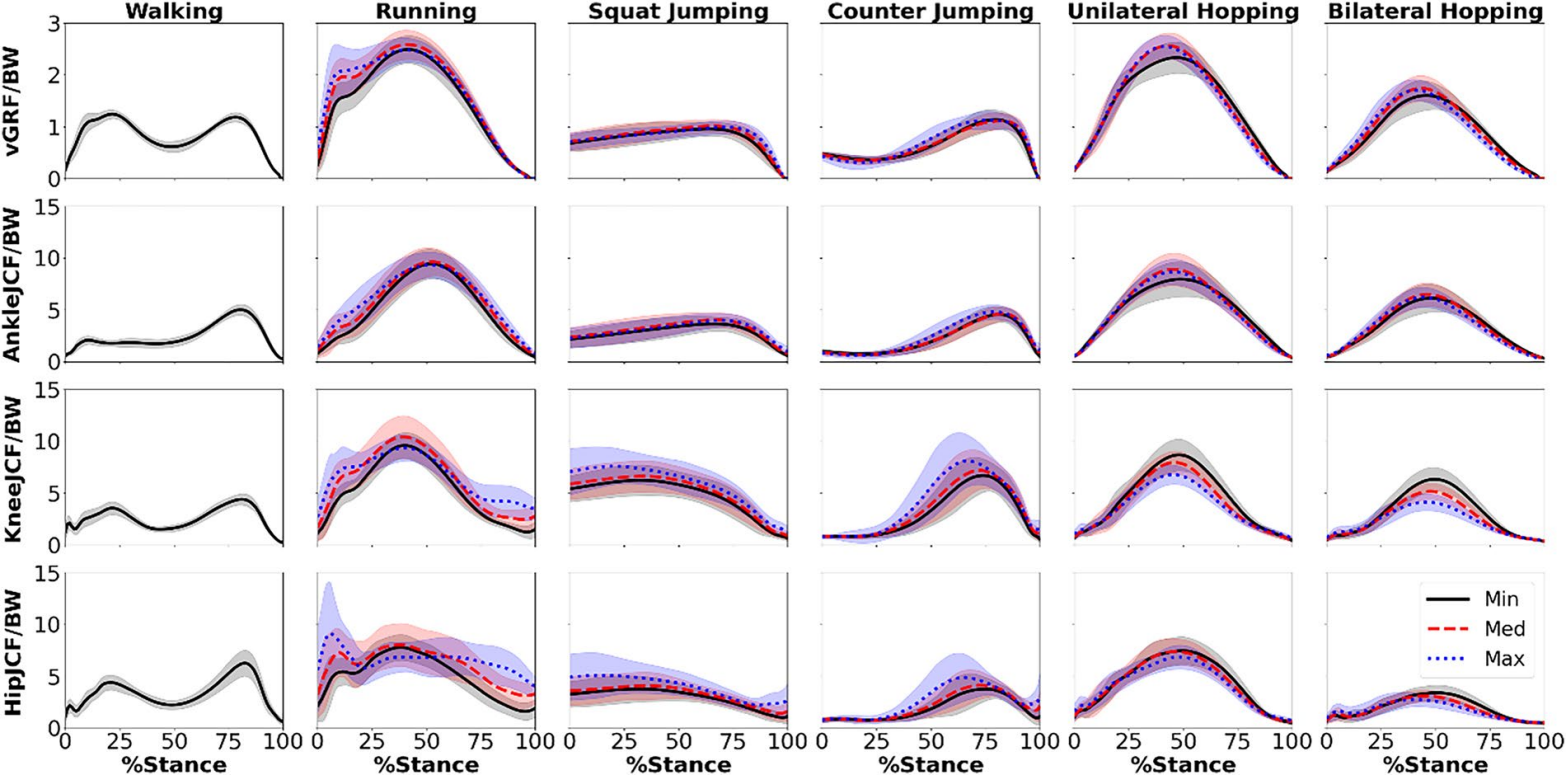
Mean vertical ground reaction force (vGRF) and joint contact force (JCF) normalised to body weight (BW). The shaded regions represent the minimum and maximum values while the black trace represents a mean JCF across all participants and trials. The data was time-normalised to 101 points during stance phase of walking and running, lowest position before the flight phase until both feet left the force plates for squat jumping, from initial stance until both feet left the force plates for countermovement jumping, and during foot contact for unliteral and bilateral hopping.

Table 4 shows the average peak (mean ±1 standard deviation) vGRF, JCF, and strains while Fig. 7 shows the vGRF and JCF curves across all trials for each exercise and intensity level. To verify the MSK model’s JCFs, comparisons were made against prior studies. For instance, vertical GRF during the stance phase of walking exhibits a dual-peak pattern and maximum of ~2.9 times body weight (BW) at the end of terminal stance^31^. Xiong *et al*. reviewed 21 studies showing that higher contact stresses occur with increased walking and running speeds^31^. The review paper also mentioned how altered biomechanics, body weight and gait characteristics can influence force outputs^31^. Additionally, this review showed how MSK modelling can validate how peak hip contact forces during stance may range between 2 to 3 times body weight during weight acceptance and push-off phases. During running, the stance phase is shorter but the peak hip joint contact force is significantly higher. Giarmatzis *et al*. found that peak forces during running can reach 7.49 times body weight, occurring at approximately 0% to 30% of the gait cycle (early to mid-stance)^32^. Muscle forces from the gluteus medius, maximus, and iliopsoas play a major role in generating these high forces during running, especially in the first half of stance^33^. Models and *in vivo* data also suggest that increasing running speed further elevates the hip joint contact force, sometimes exceeding the thresholds noted above^32,33^. The GRF and JCF values shown in Fig. 7 align with these literature values based on the qualitative patterns of the plotted data.

**Table 4:** Average peak (mean ± standard deviation) of the vertical ground reaction force and joint contact force normalised to body weight; Abbreviations: vGRF = vertical ground reaction force; JCF = joint contact force; e1 = first principal strain; e3 = third principal strain.

Exercise	vGRF (N)	JCF hip (N)	e1 (µε)	e3 (µε)
Walking	1.28 ± 0.09	6.31 ± 1.23	1985 ± 802	5053 ± 181
Running Natural (slow)	2.51 ± 0.26	8.21 ± 1.32	3389 ± 1321	8613 ± 2975
Running Moderate	2.62 ± 0.27	9.47 ± 2.17	3534 ± 1197	9127 ± 2972
Running Fast	2.62 ± 0.30	11.56 ± 4.1	3731 ± 1472	9541 ± 3610
Squat Jumps Min	1.05 ± 0.15	4.06 ± 1.33	1132 ± 744	2476 ± 1092
Squat Jumps Med	1.08 ± 0.14	4.47 ± 1.45	1302 ± 825	3003 ± 1310
Squat Jumps Max	1.03 ± 0.11	5.83 ± 2.21	1664 ± 935	3977 ± 2071
Counter Jumps Min	1.22 ± 0.18	4.18 ± 1.52	1164 ± 935	2715 ± 1148
Counter Jumps Med	1.18 ± 0.13	4.59 ± 1.44	1392 ± 769	3377 ± 1742
Counter Jumps Max	1.19 ± 0.14	5.63 ± 2.38	1693 ± 1072	4190 ± 2470
Unilateral Hopping Min	2.36 ± 0.27	7.65 ± 1.43	3003 ± 1189	7755 ± 3040
Unilateral Hopping Med	2.59 ± 0.25	7.56 ± 1.46	2729 ± 1112	7076 ± 2858
Unilateral Hopping Max	2.59 ± 0.22	6.98 ± 1.14	2439 ± 1061	6342 ± 2473
Bilateral Hopping Min	1.63 ± 0.29	3.50 ± 0.74	935 ± 459	2344 ± 1090
Bilateral Hopping Med	1.76 ± 0.24	3.18 ± 0.57	795 ± 377	2050 ± 826
Bilateral Hopping Max	1.73 ± 0.22	2.93 ± 0.76	723 ± 407	1878 ± 926

Compared to the existing datasets, the proposed dataset shows reasonable comparisons of JCF and strain values. The observed kinematic patterns combined with increased GRF and strain outputs reinforce the dataset’s biomechanical validity. For instance, our dataset shows walking JCFs peaking at ~6.3 N/kg which is comparable with prior estimates when normalised to body weight^34^. During fast running, Giarmatzis *et al*. reported peak hip JCFs reaching up to 7.5 BW, which aligns with our observed values peaking at ~8.2 N/kg^32^. Additionally, peak GRF ranged from 2.05 to 2.47 BW during running speed of 6 km/h, 9 km/h, and 12 km/h which is consistent with the peak vGRF of observed during slow, moderate, and fast running in our dataset^32^. This suggests the MSK model and optimization pipeline produced physiologically plausible results across gait tasks.

### Joint angles

**Fig. 8:**
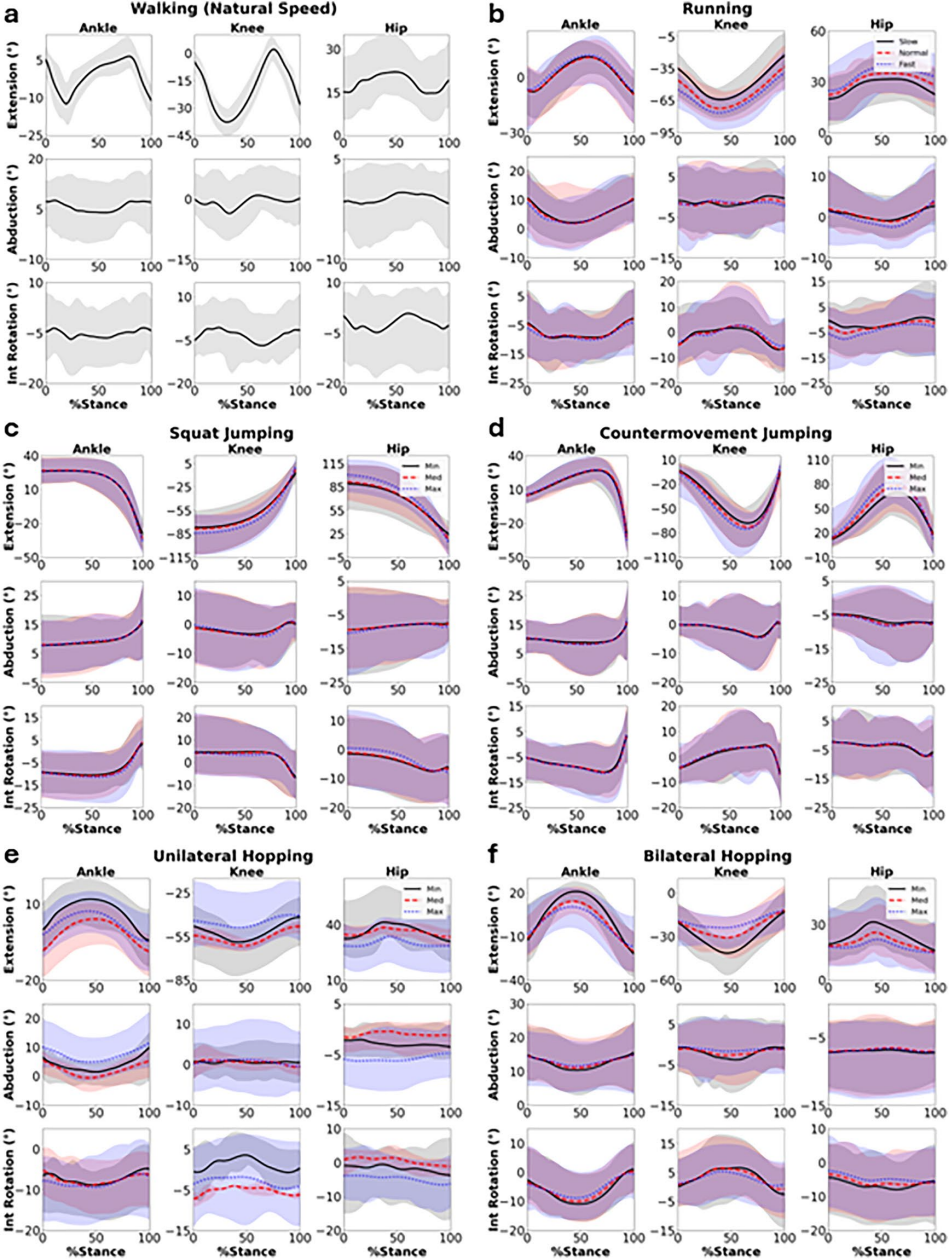
3D joint angles across participants. Solid, dashed, and dotted lines represent mean values while the shaded regions represent the minimum and maximum values across the respective intensity levels. Although the minimum and maximum shaded regions appear to have high variability, they are within the typical ranges of motion for healthy individuals involving these movements. Walking (**a**) trials had one intensity level (e.g., natural speed) while the other activities had three intensity levels. The time of interest for all trials were segmented and time normalised to 101 data points as follows: Walking (a) and Running (**b**) at stance phase of the gait cycle. Squat jumping (**c**) from the lowest pelvis position before take-off to initial take-off, and countermovement jumping (**d**) from the initial stand just before take-off to initial take-off. Unilateral hopping (**e**), and bilateral hopping (**f**) from foot contact to foot off the force plate. Int Rotation = internal rotation; Min = minimum; Med = median; Max = maximum.

Figure 8 visually demonstrate how varying intensities alter joint angles. For instance, at faster hopping speeds, joint stiffening takes place to reduce ground contact time due to the stretch-shortening cycle (SSC). Therefore, the peak joint flexion angles decreased for the ankle, knee and hip are greater during the stance portion of faster hopping compared to slower hopping movements as increased joint deformation occurs as ground contact time increases. During squat jumping and countermovement jumping, peak flexion angles increased as the intensity increases, following the literature for changes in angles and joint moments during squat jumps and countermovement jumps^35,36^. Similar trends occur during the walking and running as gait speed increases.

## Usage Notes

We have provided a README file within the Figshare folder to guide the reader on how to use the dataset^20^. This dataset provides biomechanical data during a variety of dynamic tasks and intensities which can be used to replicate the estimation of femoral neck strains from IMU signals. Furthermore, the combination of motion capture, joint contact forces, and joint angles offers potential uses for training machine learning and deep learning models to predict femoral neck strains^37^. Furthermore, clinicians and scientists can use the dataset to compare patient biomechanical patterns against normative values or predict kinetics from IMU signals. It is important to note that the MATLAB files only include the analysed hops (e.g., 4^th^ trial). Additionally, three participants were unable to complete three successful hopping trials, and therefore only two trials were recorded for them.

There are several limitations to consider when using this dataset. First, only healthy adults were included which limits the generalizability to clinical or pathological populations. Therefore, additional data on diverse populations is needed to ensure other populations are adequately represented. Second, this study was performed in a controlled laboratory environment, so careful consideration is needed prior to applying this dataset to real-world environments. Additionally, FE strain values were simulated and did not directly measure *in vivo* strains. Therefore, the assumptions of the FE models regarding bone geometry, material properties, and load estimates may alter the accuracy of strain outputs. These modeling assumptions may also influence the absolute magnitude of the predicted femoral neck strains. As such, the most appropriate interpretation of the results should be made in a relative context. For example, strain magnitudes should be compared relatively across activities (e.g., relative to walking), rather than relying on absolute values alone. Although direct *in vivo* validation of femoral neck strain during dynamic activities is not currently feasible due to ethical and technical limitations^7^, the FE modelling approach used to estimate femoral neck strain has been widely validated and applied in previous studies^38^. Also, only three repetitions per condition were performed so additional repetitions could improve the applicability of the dataset, particularly given that prescribed exercises programs, whether for performance or rehabilitative purposes tend to include sets with a higher number of repetitions^39^. Therefore, users of this dataset should consider these limitations when applying it to other contexts. Future considerations to build upon this work include training personalized machine learning models to predict bone load without the need of MSK-FE modelling. Overall, this dataset enables the scalability for robust load-based monitoring in clinical and sport settings. As a result, patients can receive tailored exercises programs aimed at reducing the risk for bone fracture and offering practical insights into bone health.

## Data Availability

Users can access the dataset publicly via Figshare^20^ using the following link: 10.6084/m9.figshare.30225739. Users are encouraged to cite the Figshare data record when using the dataset in future research.

## Code availability

A custom Jupyter notebook was used to plot the joint angles, ground reaction forces, and joint contact forces for technical validation and can be found in the following GitHub repository: https://github.com/ZainabAltai/BoneNet-A-Framework-for-Rapid-and-Accurate-Prediction-of-Femoral-Neck-strains-from-Exercise-Data. Users can download the repository to reproduce the technical validation plots.
